# Contraceptive Method Mix: Updates and Implications

**DOI:** 10.9745/GHSP-D-20-00229

**Published:** 2020-12-23

**Authors:** Jane T. Bertrand, John Ross, Tara M. Sullivan, Karen Hardee, James D. Shelton

**Affiliations:** a Tulane University School of Public Health and Tropical Medicine, New Orleans, LA, USA.; b Independent consultant, New Paltz, NY, USA.; c Knowledge Management Programs, Johns Hopkins Center for Communication Programs, Baltimore, MD, USA.; d What Works Association, Arlington, VA, USA.; e Independent consultant, Boyds, MD, USA.

## Abstract

**Context:**

Improving contraceptive method choice is a goal of international family planning. Method mix—the percentage distribution of total contraceptive use across various methods—reflects both supply (availability of affordable methods) and demand (client preferences). We analyze changes in method mix, regional contrasts, and the relationship of the mix to contraceptive prevalence.

**Methods:**

We use 789 national surveys from the 1960s through 2019, from 113 developing countries with at least 1 million people and with data on use of 8 contraceptive methods. Two measures assess the “evenness” of the mix: method skew (more than 50% use is by 1 method), and the average deviation (AD) of the 8 methods’ shares from their mean value. Population weighted and unweighted results are compared because they can differ substantially.

**Results:**

Use of traditional methods has declined but still represents 11% of all use (population weighted) or 17% (unweighted country average). Vasectomy’s share was historically low with the exception of a few countries but is now even lower. The previous trend toward greater overall evenness in the mix has slowed recently. Sub-Saharan Africa shows a hormonal method progression from oral contraceptives to injectables to implants in a substantial number of countries. In some countries with high HIV prevalence, the condom share has increased. The leading method’s share differs by region: female sterilization in Asia (39%) and in Latin America (31%), the pill in the Middle East/North Africa (32%), and the injectable in sub-Saharan Africa (36%). Method skew persists in 30% of countries. “Evenness” of mix is not related to contraceptive prevalence.

**Conclusion:**

The marked diversity in predominant methods underscores the conclusion that no single method mix is ideal or appropriate everywhere. But that diversity across countries, coupled with the persisting high degree of extreme skewness in many of them, argues for continued concerted efforts for programs to increase method choice.

## Abstract (precis)

Trends in contraceptive method mix show that dominance of 1 method in the mix remains very common, though countries and regions throughout the world are diverse as to which method is dominant. Our analysis argues for continued concerted efforts of programs to increase contraceptive method choice.

## Key Messages

- Contraceptive method mix reflects both supply and demand.
- Recent trends include a progression in hormonal methods toward implants in sub-Saharan Africa, and where HIV is common, more condom use in some countries.
- However, dominance of 1 method in the mix remains very common, though countries and regions throughout the world are diverse as to which method is dominant.
- Our analysis argues for continued concerted efforts of programs to increase contraceptive method choice.
- There is no ideal method mix; client preferences are key.

## INTRODUCTION

A key principle in both quality of care and the broader rights-based approach to family planning is method choice. As defined by the U.S. Agency for International Development, method choice exists when^1^:

*client-centered information, counseling, and services enable women, youth, men, and couples to decide and freely choose a contraceptive method that best meets their reproductive desires and lifestyle, while balancing other considerations important to safety, correct use, or switching methods.*

Method choice is a guide for optimal delivery of family planning services. To help ensure that clients’ needs are met across time and changing circumstances, the World Health Organization in 2014 recommended that family planning programs include at least 5 types of modern contraceptive methods: barrier, short-term reversible, long-term reversible, and permanent, along with emergency contraception.^2^ Method mix is an indicator that shows the pattern of actual use. It gives the percentage distribution of use across all methods in a given country, also known as “method share.” It can be calculated either in relation to women married/in-union or to all women of reproductive age, using data from a population-based survey.

Interest in the method mix of contraceptive use goes back at least to the 1980s and early 1990s, focusing not just on the empirical patterns but also upon what might constitute an “appropriate” mix.^3^ ^–^ ^5^ Choe and Bulatao (1992) compared methods for finding an appropriate mix, based partly upon the life stage of the woman, whether before or after marriage, between births, or after the final birth.^6^ Following that, Galway and Stover (1995) published a tool online to help calculate an appropriate mix, based on users’ personal profiles, the prevailing mix, method preferences shown in surveys, and method characteristics, using Kenya as a case study.^7^

Potter^8^ (1999) argued that some mixes could become outmoded as not fitting the emerging needs of the population. That could occur when the early pattern of contraceptive supply and use persisted due to being reinforced by feedback from users and program managers, as illustrated in case studies from Brazil and Mexico.

Subsequently, Bertrand et al.^9^ directed attention to method mix in which a single method accounted for more than 50% of all use (a “skewed” mix) and its relation to the quality of a national family planning program. Related analyses with data sets covering most developing countries followed,^10^ ^–^ ^12^ giving attention to changing mix patterns and their relationship to socioeconomic correlates and to the efforts of family planning programs. Ross et al.^13^ developed a different approach; rather than looking at the skew due to a single method, it took account of the distortions in mixes across all methods: the average deviation (AD) method, which is employed below along with measures of skew.

An historic disturbance to the prevailing method mixes occurred especially in countries in east and southern sub-Saharan Africa due to the steep rise in use of the injectable method starting in the 1990–1995 period. Several analyses were conducted to trace these changes in the context of their effects upon other methods.^14^ ^–^ ^16^ Rossier and Corker^17^ reviewed the use of traditional methods in sub-Saharan Africa. Rossier and colleagues also documented the underreporting of traditional methods that can occur in surveys.^18^ Recently, the United Nations (UN) Population Division published a global review of use by method, for all women rather than married/in-union women, and with regional averages population weighted.^19^

To the extent that method choice (defined above) is an underlying principle of quality family planning service delivery in developing countries, it has important implications for an “ideal” method mix. In contrast to earlier attempts to identify an “appropriate” method mix for a population, one can argue that the “ideal” method mix occurs when all women in a given country are using their desired method, consistent with the conditions outlined for convenient method choice. However, we are unaware of any research that has attempted to measure method mix from this perspective.

Method mix reflects both supply and demand. On the supply side, method choice is optimized when the full range of contraceptives is available with close geographic access, with no stock-outs or cost barriers, with adequate counseling on the methods and on the management of side effects, and with freedom from any provider bias toward or away from particular methods. Method skew may signal that potential users have only a limited choice, based on shortcomings in the supply environment. However, the measure of skew, by itself, provides little insight into the reasons for the constraints on choice.^20^

Method mix is also influenced by demand, including individual or societal preferences. Clients’ attitudes are subject to many influences. They may seek a method because it dominates the environment of what is available in the national program, as with sterilization in India.^21^ The introduction of a new method with low cost may stimulate a demand for it, as with the implant in numerous countries in sub-Saharan Africa.^22^ Demand for a given method can be adversely affected by known side effects, health concerns, misconceptions, and rumors. Donors may influence the supply of methods by decreasing the cost and supporting training in the provision of the method (e.g., implants). Program directors and providers may also emphasize certain methods over others. The private sector can also influence the availability of methods. Cultural influences are important. They inhibit sterilization use in the Middle East partly on religious grounds; Islam, as practiced in some countries, equates sterilization with prohibited mutilation of the body. By contrast, the widespread use of female sterilization in Latin America is accompanied by societal acceptance of the method as a practical means of controlling further childbearing among women who achieve their desired family size at a young age. Women may especially dislike methods, such as the intrauterine device (IUD), that require pelvic examinations. Also, for unmarried young women in some societies, confidentiality of contraceptive adoption, combined with private practice without partner or family interference,^23^ is important to avoid stigma.

Total demand for contraception (influenced by the desired family size), as well as the method-specific demand, interact with and are mediated by the constraints in the supply environment.^24^ Finally, the relative significance of supply and demand factors on method use varies across countries and across subnational entities. All of this reminds us that a perfect method has yet to appear nor can any 1 method ever be expected to be right for all clients.

This article presents new evidence on patterns and trends in method mix, overall and by regions, as well as in selected countries, for married/in-union women of reproductive age. Overall, our aim is to provide the most current picture available but with some historical information and the entire time trend for 2 illustrative countries.

We present new evidence on patterns and trends in the method mix to provide the most current picture available.

The objectives of the article are to:

(1) Document recent changes in contraceptive method mix in developing countries
(2) Examine the dominance of specific methods by region and by country
(3) Test the relationship between evenness of method mix and contraceptive prevalence
(4) Explore the implications of method skew for program applications

## DATA AND METHODS

Data for this article come from a large compilation of national surveys prepared by the UN Population Division (UN Department of Economic and Social Affairs) in its 2019 release.^25^ The database contained 1,202 surveys, from which we retained 789, using the following criteria: the country is (1) classified by the UN definition as being in the developing world, (2) has a population exceeding 1 million, and (3) has the necessary information for contraceptive use of 8 methods: female sterilization, male sterilization (vasectomy), IUD, implant, pill, injectable, condom, and traditional methods; these 8 are the focus of the analysis. Other methods in the UN series, such as the female condom, Lactational Amenorrhea Method (LAM), vaginal barrier, and emergency contraception, appear infrequently or at zero levels in the UN compilation of surveys. Moreover, the focus on these 8 methods provides continuity with earlier publications.^26^ ^–^ ^28^ Although family planning programs and donor agencies promote modern methods of contraception, we have kept traditional methods in this analysis because its use persists in numerous countries. Also, it allows us to assess the evolution in method mix from traditional methods to modern methods (or vice versa, if that is occurring).

Half of the surveys are either Demographic and Health Surveys (DHS) (34%) or Multiple Indicator Cluster Surveys (MICS) (16%), and another 27% are listed as “national surveys” done by various agencies. The rest consist of the Contraceptive Prevalence Surveys (CPS) or Reproductive Health Surveys (RHS), largely from Latin America; the Pan Arab Project for Child Development Survey and Pan Arab Project for Family Health Survey, mainly in the Middle East; and the Performance Monitoring and Accountability 2020 (PMA2020) Surveys from 11 countries.^*^

By region, 24 countries are in Asia (including 5 in the Central Asian Republics), 23 in Latin America, 21 in the Middle East/North Africa, and 45 in sub-Saharan Africa, totaling 113. The numbers of surveys in these regions, respectively, are 223 from Asia (with 20 in the Central Asian Republics), 160 (Latin America), 120 (Middle East/North Africa), and 286 (sub-Saharan Africa), totaling 789.

Regarding timing, the 789 surveys occurred from 1963 to 2018; the median survey date was 2001. By decade, the percentages were 1960s (0.6%), 1970s (7%), 1980s (14%), 1990s (22%), 2000s (31%), and the 2010s (25%). For just the latest surveys in the 113 countries, most occurred in recent years, 51 between 2010 and 2014 and 45 between 2015 and 2017. Only 17 were conducted before 2010. For analyses across time, we have annualized the trend within each country, and in analyses of regional trends we have weighted the data by population size. We have not adjusted the regional comparisons for calendar time; the dates between the earliest and latest surveys in 1 country are not necessarily the same as in other countries; moreover, the surveys can occur at different periods during the development of the national family planning program. Finally, the earliest-latest survey comparisons give the long-term picture of change, and they avoid comparisons between surveys occurring close to each other, which can introduce atypical short-term fluctuations. Correlational analyses showed that there is essentially no relationship between the size of the gap between the earliest and latest surveys and the pace of annual changes.

In this type of cross-national analysis, one can present the data as weighted (based on the population size of each country) or unweighted (in which each country has equal weight). Both have their place. Weighted data—which give every person equal importance—are useful, for example, in calculating the number of modern contraceptive users in the 69 poorest countries in the world monitored by FP2020. These estimates appropriately reflect the disproportionate contribution of large countries. By contrast, unweighted data—which give every country equal importance—are useful in assessing progress by country, as in the case of the UN Sustainable Development Goals. Rather than choose between weighted or unweighted data, we have opted to present both in this article.

To assess mix, we employ 2 indicators. The first is “method skew,” which indicates whether any single method accounts for more than half of all contraceptive use. When that extreme share occurs, the other 7 methods are necessarily relegated to smaller shares, well below 50%. Other rules could be used (e.g., 60% in the FP2020 reports),^29^ but to be consistent with previous articles on method skew, we have retained the cutoff point at 50.

To assess method mix, we used 2 indicators: method skew and average deviation.

The second measure is the AD, which Ross et al.^30^ (2015) introduced to capture the evenness of the mix across all methods, thereby augmenting the information on skew by a single method. Since use of the 8 methods adds to 100%, the average of the 8 shares is always 12.5%, and the share of each method varies around that average. The AD measure looks at the average of the deviations to capture the spread of the shares. A large spread usually indicates that just 1 or 2 methods account for most contraceptive use and the others rather little. That again suggests a limited choice. In general, the closer each method is to the mean of 12.5%, the lower the AD value. Over time, if 1 method’s share moves closer to the mean, either from above or below it, that reduces the AD value. Depending upon the country, certain methods may take zero values in an early survey if they are severely neglected or not yet made available; that makes for a high AD value. On the other hand, the introduction of a new method can increase its share of the mix, moving it up from a zero share toward the mean of 12.5%. That would result in a decline in the AD value.

If all 8 methods had an equal share of the mix, at 12.5% each, the AD value would be zero; in practice that has never occurred. The actual AD values range from 5 to 19. Perfect evenness does not exist in any country, nor would family planning experts expect it to. Further, no AD value should be considered the “ideal”; it simply serves as an objective measure that allows one to assign a score of evenness or “balance” to the method mix of each country.

In the following sections, most averages are population weighted. The levels and changes in the mix are first calculated for each country and then averaged to obtain regional estimates.

The analysis includes the following specifics:

- For trends, we calculated the change in method mix between the earliest survey and the most recent survey conducted in each country and then determined the average change for each region.
- For the latest levels, we determined the contraceptive method mix for each region and for all countries using the most recent survey conducted in each country.
- We illustrated the long-term dynamics for changes in method mix for the 2 examples of Rwanda (1983–2015) and Ghana (1979–2013).
- We identified the 34 countries with a method skew (>50%) as of the most recent survey along with the method causing the skew.
- We obtained the distribution of countries by the AD value and examined its relationship to the maximum share of use by any method, based on the most recent surveys in all countries.
- We determined the relationship between the AD value and the contraceptive prevalence rate (CPR), based on the most recent surveys in all countries.

## RESULTS

**FIGURE 1.**
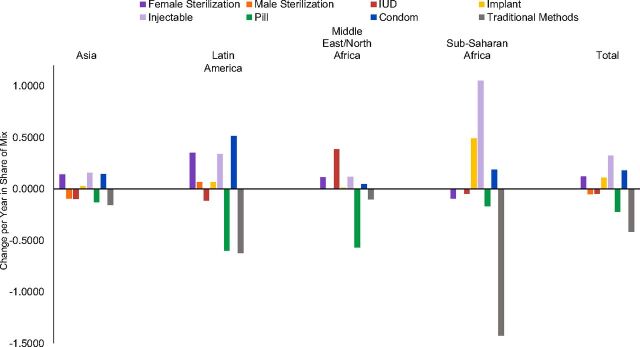
Changes in the Method Mix Between Earliest and Latest Surveys, by Method and Region, Change per Year, Weighted by Population Abbreviation: IUD, intrauterine device.

This analysis captures the dramatic changes in method mix over several decades of international family planning. Among the 113 countries studied, 109 had 2 or more surveys, allowing for changes between the earliest and the latest surveys (Supplement). The time periods varied around an average interval between surveys of 17 years. Figure 1 summarizes these changes by region and for all countries. The changes are annualized to allow for dissimilar observation periods, and they are population weighted. The bars above the line denote gains by a method; those below the line, losses. Changes within each region add to zero. For all countries, traditional methods lost an annual average of 0.42 points of share, or 4.2 points over 10 years. The pill also lost share, and small losses occurred for male sterilization and the IUD. Meanwhile, female sterilization, the implant, the injectable, and the condom gained shares.

Among regions, Asia showed the smallest changes while sub-Saharan Africa showed the most, with Latin America and the Middle East/North Africa experiencing intermediate degrees of change. The most extreme shift was in sub-Saharan Africa with the injectable replacing traditional methods. In the early years, its CPR was often low, so that traditional methods could represent a large percentage of a small pie.

As explained in the Methods and Data section, we addressed any concern about methodological differences across the survey types by rerunning the results just with the DHS and MICS surveys and found essentially no differences in the main levels and patterns. We therefore decided to use the full set of surveys to enlarge the base by regions and to augment the time trends.

### Key Changes in Method Mix in Recent Years

From this analysis, we identified 4 key trends.

#### 1. Traditional Method Use Has Declined Over Time but Remains Substantial

Traditional method use remains perplexing and somewhat controversial among international family planning experts. Some argue that programs should actively try to move clients from traditional to modern methods, given the greater effectiveness of the latter in preventing pregnancy. Others contend that traditional methods, which are “natural,” serve a valuable purpose; they are noninvasive, free, always available, and have no side effects. Some maintain that while family planning programs should not necessarily promote traditional methods, people should know how to use them (particularly withdrawal) in case they are having sex without any other method available. Still others view traditional methods as a bridge to modern contraceptive use, especially when a woman has experienced an unplanned pregnancy while using a traditional method.

Despite the tremendous strides made in family planning programming worldwide over the past 5 decades, a surprising 11% of all users, or about 1 in 10, continue to rely on traditional methods. In each country, trends in the use of each method are derived from the change between the earliest available survey to the latest one. This approach provides the experience of the country over the long term, while mitigating short-term fluctuations and measurement errors. The annual rate of change is used to allow for different observation periods between the surveys.

Despite tremendous strides in promoting modern contraceptive methods over the last 50 years, about 1 in 10 users still rely on traditional methods.

Averaging over all countries, the annual rate of decline for traditional methods has been 0.42%, or 4.2% over 10 years (Figure 1, total bar). Regional averages varied considerably, as the above examples suggest. The loss of traditional share was least in the Middle East/North Africa at only 0.10% and greatest in sub-Saharan Africa at 1.42%. The loss was quite different between Asia (a low 0.16%) and Latin America (a much higher 0.62%). Thus, the loss of share for traditional methods was considerable and quite variable by region. The large loss in sub-Saharan Africa probably reflects the high initial reliance on traditional methods, falling to lower levels as modern methods rose.

**FIGURE 2.**
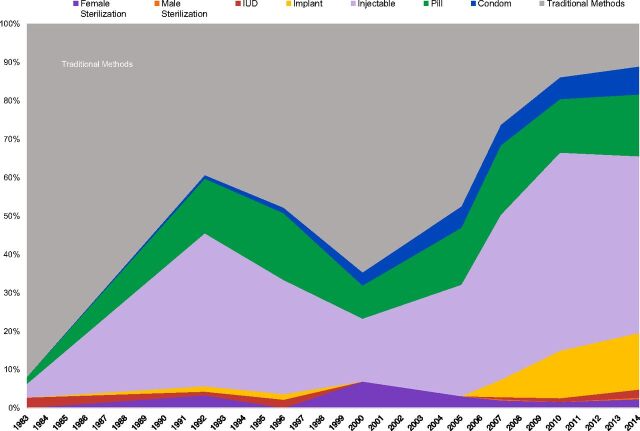
Rwanda: Changes in Method Mix Between 1983 and 2014^a^ Abbreviation: IUD, intrauterine device.^a^ In the middle of Figure 2, the share due to traditional methods increased and the shares for modern methods fell. The timing corresponds to the Rwanda genocide in mid-1994; overall contraceptive use fell from about 20% to about 13% between the surveys of 1992 and 1996 but proportionately less for traditional methods than for resupply methods dependent upon logistics systems.

**FIGURE 3.**
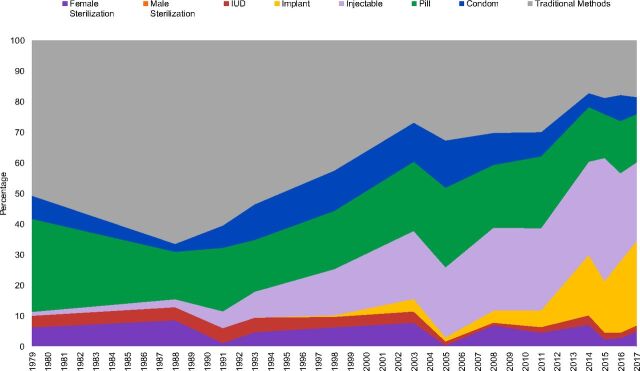
Ghana: Changes in Method Mix Between 1979 and 2017 Abbreviation: IUD, intrauterine device.

Two country examples vividly illustrate the possible changes in method mix over time. In Rwanda, the traditional share fell from 92% in 1983 to only 11% in 2015 (Figure 2), a decline of 81%, the largest on record. In Ghana, (Figure 3) the traditional share fell from 52% to 18%, a 34% decline, less than in Rwanda but down to only one-third of the starting level.

#### 2. Vasectomy’s Share of Method Mix Has Declined, From Low to Lower

Vasectomy has had limited uptake for a combination of reasons related to supply and demand, especially in recent years. In the 789 surveys examined here, vasectomy equaled or exceeded the “equal share” of 12.5% only in the Republic of Korea (all surveys 1985–2006), Nepal (all surveys 1976–2011), and Thailand (14.2% in 1969), though close to equality in China (12.1% in 1992). Relatively high values elsewhere occurred mainly in the early days of family planning programming, from the 1960s through the mid-1970s, when few other methods were available. As with traditional methods, in early programs, the percentages for vasectomy often represented a large share of quite low prevalence.

Vasectomy’s share has undergone a drastic decline in 7 countries where it was important, between the peak year of its use and the year of the most recent survey. In each country, its share of the method mix has plummeted.^**^ Here are the declines, in order of the starting levels of the shares: Nepal 47.1 to 10.5 (the highest current figure), Thailand 14.2 to 0.5; China 12.1 to 1.7; Myanmar 10.7 to 0.6; India: 8.6 to 0.6; Sri Lanka: 8.2 to 0.0; and Bangladesh 7.1 to 1.9. Most other countries in the data set showed small, non-zero percentages for vasectomy, and in no country did vasectomy increase its share over time.

Regarding national policies, a few countries have promoted the voluntary use of vasectomy with some success (for example reaching 5% of the method mix in Colombia by 2016 and in Brazil by 2013), but the method faces cultural and gender barriers, especially in sub-Saharan Africa, with concerns that men will lose their strength and masculinity if they have the procedure.^31^ Vasectomy also faces religious barriers in Muslim countries,^32^ ^,^ ^33^ as does female sterilization in most Muslim countries. However, female sterilization accounts for a quarter of all use in Pakistan, about 7% of the mix in Bangladesh, 13% in Turkey, and 18% in Iran. In any case, few programs have opted to promote vasectomy in recent years, and in practice, policy makers have shown little political will to explicitly promote vasectomy.

Modifications in the mix reflect the **relative** changes in the prevalence of the methods over time. If, for example, the use of traditional methods remains about the same while the use of modern methods increases, leading to a rise in total contraceptive use, that produces a diminishing **share** of all use for traditional methods. In India, total prevalence of use rose from 40.7% to 53.5% between the 1992/93 and the 2015 surveys. Female sterilization rose from 27.4% to 36.0%, while male sterilization declined from 3.5% to 0.3%. For the mix, that translates to a stable female share of 67.3% in both surveys and a decline in the male share from 8.6% to 0.6%.

For prevalence, overall sterilization was gaining. Vasectomy was declining, but female sterilization was increasing enough to more than compensate, and it was doing so in the context of other method changes (Figure 1). For shares that was the general pattern: in a full set of within-country comparisons, the share for female sterilization rose on average twice as fast as the male share did.

**Weighted vs. unweighted results:** The mix looks quite different when the results are weighted by the population size of the country versus unweighted, when each country has an equal weight.

Overall, in Table 1, last row, 11% of all users rely on traditional methods (weighted data), whereas the country average is higher at 17%. The difference reflects the impact of the largest countries, where fewer rely on traditional methods. Other methods also reflect the impact of the largest countries. In Asia, 39% of all users rely on female sterilization, but a mere 13% do so as the average country. The high figure is due to India’s 67% of users on female sterilization, followed by China’s 34%, which together represent two-thirds (69%) of the region’s population. Table 1 shows that the difference is reversed for the injectable: it is not important in India and China, but it is very important in Indonesia, the region’s third largest country. The total rows give the overall contrasts for each of the 8 methods, including the large difference for female sterilization.

**TABLE 1.** Method Mix for Latest Surveys to Compare Unweighted and Weighted Results

		FemaleSterilization	MaleSterilization	IUD	Implant	Injectable	Pill	Condom	Traditional	Sum
Asia	Unweighted	13.4	1.9	24.8	2.5	13.9	17.8	13.2	12.6	100.0
	Weighted	38.7	2.6	22.0	1.0	6.6	10.0	10.7	8.4	100.0
Latin America	Unweighted	29.1	1.2	8.4	1.9	16.4	19.1	14.3	9.5	100.0
	Weighted	30.8	2.6	9.2	2.5	10.7	23.2	13.2	7.8	100.0
Middle East/North Africa	Unweighted	7.2	0.3	20.2	0.2	4.6	29.4	8.6	29.5	100.0
	Weighted	7.7	0.6	23.4	0.3	5.6	32.0	8.2	22.2	100.0
Sub-Saharan Africa	Unweighted	3.9	0.2	3.4	11.3	30.8	21.4	12.0	16.9	100.0
	Weighted	3.5	0.1	3.1	13.9	35.7	18.9	7.8	17.0	100.0
Total	Unweighted	11.6	0.8	12.0	5.5	19.6	22.0	11.9	16.6	100.0
	Weighted	29.1	2.0	17.5	3.3	12.0	14.8	10.2	11.0	100.0

Abbreviation: IUD, intrauterine device.

#### 3. In sub-Saharan Africa, a Hormonal Method Progression From Oral Contraceptives to Injectables to Implants Is Evident

The sub-Saharan Africa region is especially relevant for contraceptive dynamics, as it shows the greatest amount of change as countries move toward modifications in the method mix.

Historically in sub-Saharan Africa, hormonal methods have dominated, apart from traditional methods. In the 1970s and 1980s, such use consisted largely of oral contraceptives. But with U.S. Food and Drug Administration approval of the injectable DMPA in 1994, injectables progressively became the predominant method in many countries. Implants first appeared with the approval of 6-capsule Norplant in 1990, followed by more advanced implants. Yet, provision of implants remained fairly modest, constrained both by fairly high cost and a limited service delivery infrastructure to provide them. However, price/volume guarantees negotiated between donors and the 2 major implant manufacturers in 2012 and 2013 reduced the price dramatically.^34^ Moreover, improved service delivery mechanisms, notably mobile service delivery and social franchising, vastly increased implant availability. The high and increasing prevalence of the implant (and its percentage of market share) is due not only to high adoption rates, but to the long continuation of use that the implant offers. However, after the recommended period of use, removals and reinsertions are needed, so a result of the growing numbers of users is that implant removals will accelerate, as noted by Christofield and Lacoste.^35^

A good example of hormonal progression is Ghana. The leading method in the 1970s and 1980s was the oral contraceptive; it was overtaken by the injectable in the mid-2000s, which in turn was overtaken by the implant by 2017 (Figure 3). Currently, the shares are pill, 16%; injectable, 26%; and implant, 28%. The general hormonal progression pattern is evident in at least 21 other countries: Angola, Benin, Burkina Faso, Burundi, Chad, Ghana, Guinea, Guinea Bissau, Liberia, Malawi, Mali, Niger, Nigeria, Rwanda, Senegal, Sierra Leone, Timor Leste, Tanzania, Togo, Uganda, and Zambia.

The latest entry in hormonal method choice is subcutaneous injectable DMPA or DMPA-SC.^36^ It provides a lower dose of DMPA in an approach that is more conducive to community service delivery and even to self-injection. DMPA-SC is already becoming popular in several African countries due partly to the self-injection option.^37^

#### 4. Condom Use for Contraception Has Increased in Some Countries With High HIV Prevalence

Worldwide, HIV prevalence is highest in sub-Saharan African countries.^38^ Not surprisingly, with the advent of HIV, condom use has risen to substantial shares of all contraceptive use in some of those countries. For Botswana, Lesotho, and eSwatini (formerly Swaziland), condoms are the first or second most widely used contraceptive method; their shares of the method mix are 69%, 37%, and 28%, respectively. Several other countries have relatively high condom shares: Angola (23%), Namibia (22%), and South Africa (16%). In contrast, in other countries, the condom method share is only in the single digits: Zimbabwe (6%), Mozambique (6%), and Malawi (3%.) It is likely that condom use is higher than these figures indicate, since some women are reluctant to admit condom use; also, when 2 modern methods are reported including the condom, the rule is to classify such clients only under the other modern method.

### Method Mix and Skew According to Region and Country

#### 1. The Predominant Method Differs by Region

**FIGURE 4.**
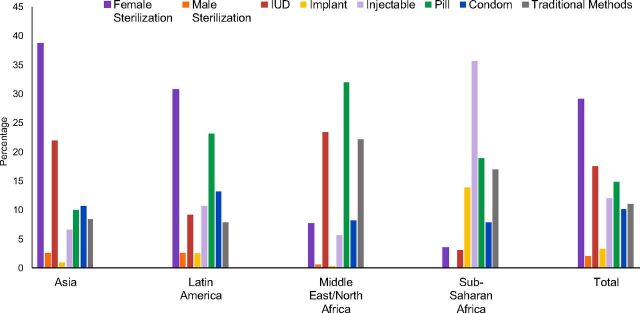
Contraceptive Method Mix in Each Region and All Countries, Population Weighted Abbreviation: IUD, intrauterine device.

For all countries, as noted above, the most widely used method is female sterilization (29%), followed much lower by the IUD (18%). The pill (15%), injectable (12%), traditional methods (11%), and condom (10%) follow. The smallest percentages correspond to implants (3%) and vasectomy (2%) (Figure 4, total bars, and Table 1, weighted totals).

This overall perspective masks the remarkable fact that the leading methods differ considerably by region (weighted data) and country: female sterilization in Asia (39%) and in Latin America (31%), the pill in the Middle East/North Africa (32%), and the injectable in sub-Saharan Africa (36%). Within individual countries, the shares vary quite widely.

Why these sharp disparities? The share of each method reflects each region’s own balance of supply and demand influences over time. The sterilization share builds up gradually from annual adoptions over past years, during which those influences would have changed; the same is true for the other long-acting methods of the IUD and implant. On the other hand, current users of the resupply methods (condoms, pills, injectables) come largely from adoptions in the recent past since their average use time is relatively short; therefore, their use is more sensitive to recent influences, such as supply interruptions and shifting method preferences. Disparities in the family planning environment are large and fundamentally different in countries as dissimilar as India and Mali, and the result is a blend of cultural background, donor involvement, provider priorities, cost, access, and public response to the methods offered. In general, there is variety in pattern but consistency in a region over time.

The marked differences in method mix reflect each region’s own balance of supply and demand influences over time.

#### 2. Method Skew Persists Over Time, but the Evenness of Method Mix Varies Greatly by Country

The number of countries with method skew has remain unchanged in recent years. Evidence from the most recent surveys shows that in these 113 countries, 34 countries (or 30%) show a skewed method mix, the same as the 30% found by Bertrand et al.^26^ and slightly lower than the 35% reported by Sullivan et al.^27^ In short, close to a third of developing countries still have a skewed method mix.

In the 34 countries with method skew, the leading method differs considerably. As shown in Table 2, the number of countries skewed toward each method is injectable (9), traditional methods (7), pill (7), IUD (6), female sterilization (3), and condom (2). Table 2 also shows the extent of method skew in each country. In no country does male sterilization or the implant have a share more than 50%, although the share for the implant has reached 46% in Burkina Faso. Also noteworthy, in half (17) of the countries, the method skew exceeds 60%.

**TABLE 2.** The 34 Countries That Have a Method Skew (>50%) as of the Most Recent Survey and Method Causing the Skew, Based on Women Married or in Union

**Method**	**Country**	**Skew (%)**
Injectable	Ethiopia	64.4
	Liberia	62.8
	Haiti	61.7
	Sierra Leone	54.3
	Myanmar	52.9
	Mozambique	51.9
	Indonesia	51.8
	Madagascar	51.1
	Malawi	50.8
Traditional	Azerbaijan	76.8
	South Sudan	65.7
	DR Congo	64.8
	Armenia	51.9
	Libya	51.6
	Bahrain	51.3
	Mauritius	50.7
Pill	Sudan	77.6
	Algeria	77.5
	Morocco	74.7
	Saudi Arabia	62.0
	Zimbabwe	61.7
	Mauritania	59.8
	Laos	50.6
IUD	Turkmenistan	87.5
	Uzbekistan	80.0
	Tajikistan	64.4
	Kyrgyzstan	55.6
	Kazakhstan	54.4
	Egypt	51.5
Female Sterilization	India	67.7
	Dominican Rep.	58.6
	El Salvador	51.7
Condom	Hong Kong	70.0
	Botswana	69.3

Abbreviation: IUD, intrauterine device.

Returning to the AD values as a measure of the evenness of the mix, we find that the 113 countries follow a bell-shaped curve, with a roughly normal distribution. Around the AD median of 11.8, about half of countries (65) are in a middle range, falling between ADs of 9.9 and 13.7, and 97 are within the wider range of ADs 8.6 to 15.0. A few are at relatively extreme values; for example, Nepal in the low range with an AD of only 6.6, and Egypt in the high range with an AD of 14.0. Those in the high range contain the especially skewed cases.

### Total Contraceptive Prevalence Is Not Related to the Evenness of the Mix

**FIGURE 5.**
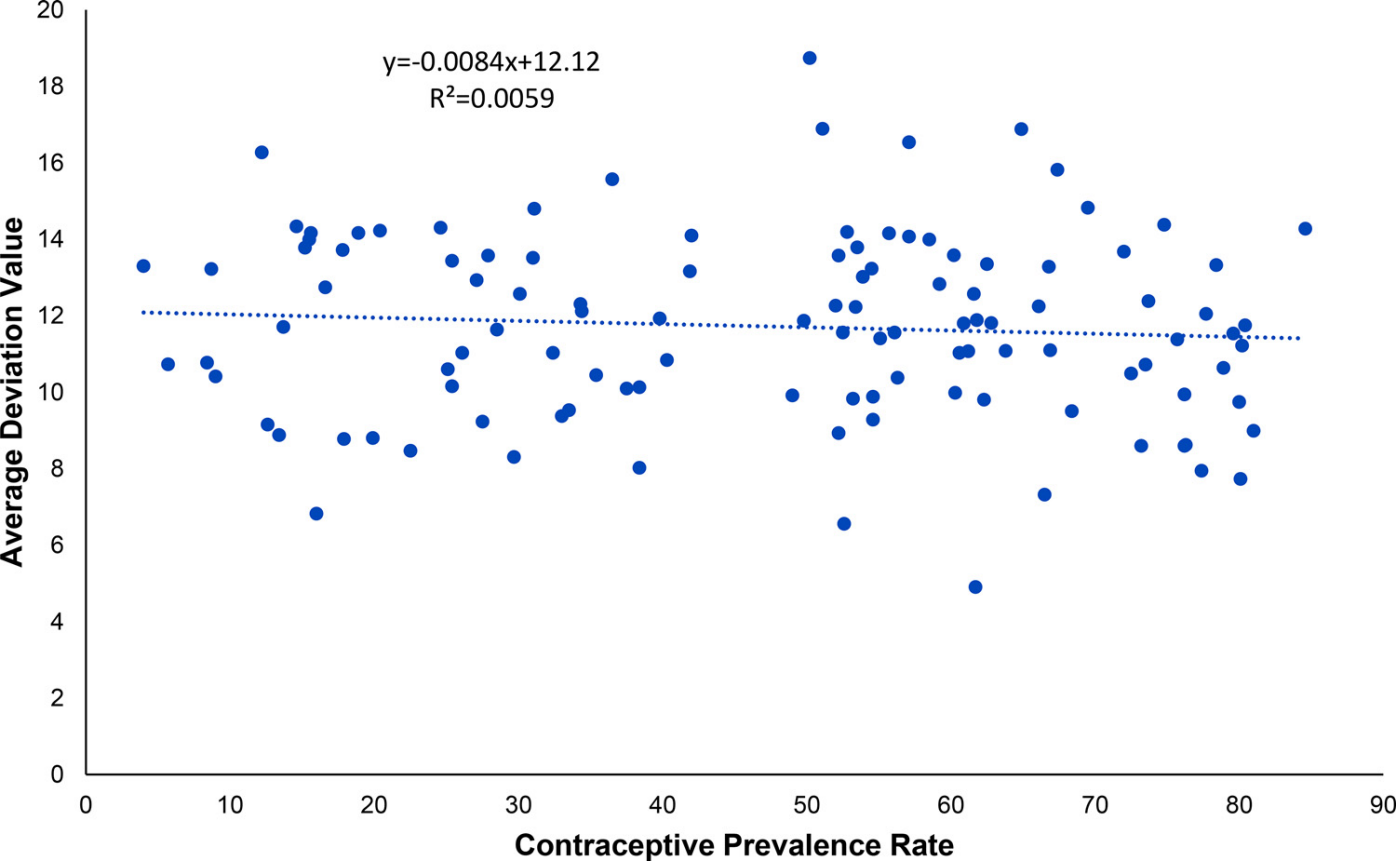
Relationship Between the Measure of Average Deviation and Contraceptive Prevalence Rate, 113 Latest Surveys

Previous research has indicated that increasing the number of available methods results in higher contraceptive prevalence,^39^ but that can either increase or decrease the evenness of the mix. Based on the 113 most recent surveys, we found no statistically significant relationship (R2=0.0065, *P*=.95) between the evenness of method mix as measured by the AD and contraceptive prevalence (Figure 5). As the CPR rises, the AD values do not systematically change. There is a large variation in the AD values at any level of the CPR.

Several reasons appear to account for this lack of association. First, some countries, such as China or Vietnam, with high CPRs rely on only 1 or 2 modern methods, showing a highly skewed method mix. Other countries, such as Niger and the Democratic Republic of the Congo, are also highly skewed, but at low CPR levels. Additional countries at middle CPRs vary considerably in the spread of their methods, some with narrow spreads and others with wide ones. All this reflects regional disparities in method access and choice as well as other factors.

## DISCUSSION

This analysis shows at least 3 positive trends: a decline in the shares held by traditional methods in favor of more effective contraceptives, a “hormonal progression” in sub-Saharan Africa with countries moving from pills to injectables and in many cases on to widespread implant use, and the increased use of condoms in some countries with high HIV prevalence. Yet, challenges remain. Despite more than 5 decades of international family planning, traditional methods represent an average of 17% of the method mix in the 113 countries analyzed, or 11% of all users. And close to a third (30%) of countries still report method skew, with over half of all use by a single method.

Two unexpected findings are that a more even method mix is not associated with a higher CPR, and that the leading contraceptive methods differ considerably more among regions than we would have anticipated.

The current mix is a function of 2 dissimilar dynamics: use of the long-term methods is an accumulation of adoptions over past years, whereas use of short-term methods comes from recent starts, due to their shorter continuation rates. Therefore, the impact of current program initiatives and other determinants of use can be considerably greater among the short-term methods.

Some countries have implemented deliberate measures to diversify method mix. An intensive effort in parts of 5 crisis-affected countries (Chad, the Democratic Republic of the Congo, Djibouti, Mali, and Pakistan) to widen access to several methods resulted in 61% of clients selecting implants and IUDs.^40^ In Indonesia, community-led advocacy efforts implemented in the 6 Improving Contraceptive Method Mix project districts yielded increases in uptake of long-acting and permanent contraceptive methods, against a national context in which about half of users rely on the injectable.^41^ Yet, elsewhere such initiatives have failed to change the mix, such as efforts in Morocco in the 1990s to increase IUD use in a “pill” country.^26^ Despite efforts to encourage the uptake of vasectomy, its use has fallen sharply wherever it had claimed a significant share of use; currently the highest share is 10% in Nepal, 5% in Brazil and Colombia, and close to zero in many developing countries.

What explains the persistence of method skew in some countries? The 34 countries we found with skew are nearly the same as those in the 2006,^27^ 2014,^26^ and 2015^30^ reviews. Method mix is like a slow-moving ship: it is possible to change direction only over time. It is often difficult to disentangle the 2 main categories of factors that influence skew: limitations on the supply side (lack of access to a wider range of contraceptives, beset by stock-outs, cost barriers, and provider biases) versus those on the demand side, including ingrained societal preferences. Is the high level of female sterilization in India or the Dominican Republic the result of constrained supply of alternative methods, normatively influenced demand, or both?

Method mix is like a slow-moving ship: it is possible to change direction only over time.

The above analyses allow us to better understand the current status of method mix, its evolution over time, and its diversity by region and country. Yet, key questions remain. First, to what extent is continuous method skew a problem in countries with high CPRs? Numerous countries have CPRs above 60% and are skewed by the 50% rule: Dominican Republic, Mexico, and El Salvador for female sterilization, Morocco and Zimbabwe for the pill, and near cases for the IUD: China and Vietnam with 48% of use on the IUD. We are unaware that any of these countries are taking action to improve the evenness of the mix.

Second, is it really a problem if a country moves toward greater method skew after the introduction of a new method, if the method enlarges choice and helps meet the needs of clients? For example, in Burkina Faso 46% of users now rely on implants, and other sub-Saharan African countries are moving in this direction.

Third, in the absence of an “optimal” or “ideal” method mix, are there measures that better capture the balance in contraceptive method mix that some program managers and donors seek and that are believed to better meet clients’ diverse needs? Bertrand et al.^9^ proposed using the real-life experience of countries that come closest to having a fully balanced method mix and also have at least a moderately high CPR, defined as 25%. Yet, in the absence of a widespread initiative to improve method mix, any method to improve the measurement of “balance” in method mix seems to lack programmatic relevance.

Another approach would be to examine possible relationships between family planning program effort measures^30^ and the characteristics of the mix. If strong programs best service the needs of clients, the resulting mix may be closer to a preferred standard. Such work would need to take into consideration the vast divergence among regions in predominant methods.

### Limitations

Regarding limitations in this work, one relates to the surveys available. The number of surveys per country varied from 1 to 18, which decreased the sensitivity of the time trends in countries with few surveys. Also, the surveys were not conducted in the same years or at a constant interval, and we included multiple types of surveys (e.g., DHS, MICS, CPS, PMA2020) with their dissimilar methodologies. However, concerns about the latter were allayed by the reruns done with only the DHS and MICS types, which gave very similar results to those produced by the full set.

Our primary focus on method mix resulted in less attention to prevalence. In countries where total prevalence is quite low, the mix among the 8 methods is less stable over time, and the share estimates are subject to greater sampling error. Total prevalence has risen in many countries, so that a method can lose share and still keep the same level of prevalence. Historically, countries have moved for example from a high share of traditional methods toward lower shares, even while the absolute level of their prevalence may have changed little. Wanting to focus especially on trends in the mix both overall and by regions, we gave less attention to the complexity of method mix as it occurs in particular countries. Nor did we analyze the relationship between method mix and economic status of countries by their GDP per capita or similar measures.

We did not undertake a separate analysis of method availability as a determinant of the mix, as beyond our scope. Measures of availability are found in the FP2020 annual report for the 69 poorest countries in the world^42^ and in a study of national family planning program efforts in more than 80 developing countries.^43^ Any analysis of the relationship of availability to other measures must contend with the problem that data are not always available for the year corresponding to the latest nationally representative survey; moreover, “availability” has several dimensions including geographic access, cost, and quality of care at the source of each method.

## CONCLUSION

A future step in researching method mix involves more in-depth analysis of the methods that produce the unevenness in method mix in relation to total contraceptive prevalence. Our analyses do not address the complex relationships among choice, total prevalence of use, and the various mix patterns. Most use in most countries is accounted for by 2 to 3 methods. Limited choices only partly account for that since consumer preferences enter in, and a full choice of many methods might not alter the prevailing pattern. Nevertheless, past experience confirms that the addition of more methods to a narrow mix increases prevalence, up to some limit. Further work into the history of which methods and at what prevalence levels would be of interest.

## Supplementary Material

20-00229-Bertrand-Supplement.pdf

## Footnotes

* The full list of surveys used in the UN series included: Caribbean Contraceptive Prevalence Survey; Contraceptive Prevalence Survey; Demographic and Health Survey; Gulf Child Health Survey; Gulf Family Health Survey; Generations and Gender Survey (Rep. of Georgia); Living Standards Measurement Study survey (Pakistan); Multiple Indicator Cluster Survey; National survey that is not part of a multi-country survey program; Pan-Arab Project for Child Development Survey; Pan-Arab Project for Family Health Survey; Performance Monitoring and Accountability 2020 Survey; Reproductive Health Survey; and World Fertility Survey.

** The shares were declining even while the prevalence values were rising in Bangladesh and Nepal. The underlying prevalence values (percentages) for the shares listed were: Bangladesh: 0.9 to 1.2; China: 10.2 to 1.4; India: 3.5 to 0.3; Myanmar: 1.8 to 0.3; Sri Lanka: 3.9 to 0.0; Nepal: 1.6 to 5.5; and Thailand: 2.1 to 0.4.

## Notes

**Peer Reviewed**

**First published online:** November 6, 2020
