# Supplementary material for: Contraceptive Method Mix: Updates and Implications
Source: Glob Health Sci Pract. 2020 Dec 23;8(4):666–79. doi: 10.9745/GHSP-D-20-00229 (PMC7784075; doi:10.9745/GHSP-D-20-00229)
Supplement: 20-00229-Bertrand-Supplement.pdf [file 20-00229-Bertrand-Supplement.pdf]

Supplement. Method Mix for the Earliest and Latest Surveys in 113 Countries										
Note: Only one survey for The United Arab Emirates and Venzuela										
		Female Steril	Male Steril	IUD	Implant	Injectable	Pill	Male condom	Traditional methods	Sum
ASIA										
Afghanistan	2000	16.3	2.0	6.1	0.0	26.5	24.5	0.0	24.5	100
Afghanistan	2015.5	8.5	0.0	6.6	0.9	23.2	32.2	15.6	12.8	100
Bangladesh	1975.5	7.8	6.5	6.5	0.0	0.0	35.1	9.1	35.1	100
Bangladesh	2014	7.4	1.9	1.0	2.7	19.9	43.3	10.3	13.5	100
Cambodia	1995	10.4	0.0	12.8	0.0	18.4	10.4	2.4	45.6	100
Cambodia	2014	5.3	0.2	7.8	3.9	16.2	31.7	3.7	31.1	100
China	1982	25.3	9.9	50.2	0.0	0.0	8.5	2.0	4.0	100
China	2006	34.0	5.3	48.1	0.4	0.0	1.4	10.1	0.7	100
Hong Kong	1982	28.5	1.7	5.0	0.0	3.9	27.8	20.9	12.2	100
Hong Kong	2012	4.7	0.3	8.6	0.0	2.2	10.9	70.0	3.4	100
India	1980.5	0.0	0.0	3.8	0.0	0.0	7.7	32.3	56.2	100
India	2015.5	67.7	0.6	2.8	0.0	0.0	7.7	10.5	10.7	100
Indonesia	1976	1.1	0.0	21.4	0.0	0.8	56.9	6.9	13.0	100
Indonesia	2016.5	6.1	0.0	7.2	8.6	51.8	20.4	2.3	3.6	100
Korea, Rep.	1965	0.0	6.3	25.0	0.0	0.0	6.3	50.0	12.5	100
Korea, Rep.	2009	8.2	23.4	17.9	0.0	0.0	2.8	33.9	13.8	100
Laos	1993	27.6	0.0	6.5	0.5	11.4	34.6	0.5	18.9	100
Laos	2011.5	9.3	0.0	3.2	0.2	27.6	43.0	2.2	14.4	100
Malaysia	1974	10.3	1.2	2.1	0.0	0.6	49.2	8.2	28.3	100
Malaysia	2014	13.3	0.0	5.2	1.3	9.4	25.4	10.8	34.5	100
Mongolia	1994	1.6	0.0	57.7	0.5	1.9	4.4	5.9	28.0	100
Mongolia	2013	5.9	0.0	43.2	0.9	6.8	16.0	15.4	11.8	100
Myanmar	1991	22.0	10.7	5.4	0.0	18.5	23.8	0.6	19.0	100
Myanmar	2015.5	9.2	0.6	5.4	1.7	52.9	26.4	1.9	1.9	100
Nepal	1976	2.9	47.1	2.9	0.0	0.0	11.8	5.9	29.4	100
Nepal	2016.5	28.1	10.5	2.7	6.3	17.0	8.8	8.0	18.7	100
Pakistan	1975	17.3	1.9	13.5	0.0	0.0	19.2	19.2	28.8	100
Pakistan	2012.5	25.7	0.9	6.8	0.0	8.3	4.7	26.0	27.5	100
Philippines	1978	13.1	1.9	6.4	0.0	0.6	12.5	10.0	55.6	100
Philippines	2013	15.6	0.2	6.4	0.0	6.8	35.0	3.5	32.5	100
Sri Lanka	1975	0.0	0.0	21.8	0.0	1.7	7.1	9.7	59.7	100
Sri Lanka	2016	22.4	0.0	16.4	7.2	13.2	13.2	10.7	16.9	100
Thailand	1969.5	37.2	14.2	14.9	0.0	2.7	25.7	5.4	0.0	100
Thailand	2015.5	31.0	0.5	0.5	1.4	17.9	41.9	3.2	3.6	100
Timor-Leste	1991	0.8	0.0	17.9	14.3	41.8	5.6	0.8	18.7	100
Timor-Leste	2016	5.4	0.0	7.7	23.9	45.2	8.5	0.0	9.3	100
Viet Nam	1988	5.1	0.6	62.3	0.0	0.0	0.8	2.3	29.0	100
Viet Nam	2015	2.2	0.1	48.0	0.4	1.7	18.8	14.7	14.1	100
**CENTRAL ASIA REPUBLICS**										
Kazakhstan	1995	0.0	0.0	68.2	0.0	0.0	3.1	6.4	22.4	100
Kazakhstan	2015	3.1	0.0	58.6	0.0	0.2	11.2	23.0	3.9	100
Kyrgyzstan	1997	3.0	0.0	64.3	0.0	2.2	2.9	9.6	18.0	100
Kyrgyzstan	2014	3.2	0.0	55.6	0.0	0.5	10.2	25.8	4.7	100
Tajikistan	2000	0.6	0.3	76.3	0.0	2.7	1.8	1.2	17.0	100
Tajikistan	2012	2.2	0.0	66.8	0.0	7.2	8.3	7.9	7.6	100
Turkmenistan	2000	3.4	0.0	72.6	0.0	1.9	2.2	3.7	16.2	100
Turkmenistan	2015.5	0.6	0.2	87.5	0.0	0.6	2.0	3.0	6.2	100
Uzbekistan	1996	1.3	0.0	82.4	0.0	2.5	3.1	3.1	7.7	100
Uzbekistan	2006	3.4	0.2	80.0	0.2	4.3	3.7	3.4	4.8	100

**LATIN AMERICA**										
Argentina	2001	0.0	0.0	12.6	0.0	0.0	40.3	29.5	17.6	100
Argentina	2004.5	7.4	0.0	10.6	0.0	3.0	25.9	41.7	11.4	100
Bolivia	1983	10.4	0.4	14.7	0.0	1.7	11.7	1.7	59.3	100
Bolivia	2016	15.1	0.2	10.6	5.9	18.6	6.1	10.6	33.1	100
Brazil	1986	40.7	1.2	1.5	0.0	0.9	38.3	2.6	14.7	100
Brazil	2013	26.8	5.3	2.5	0.1	6.6	42.8	12.9	3.1	100
Chile	2001	9.4	0.0	35.9	0.0	1.0	38.5	10.7	4.5	100
Chile	2015.5	15.9	0.1	22.8	0.0	9.2	33.3	10.1	8.5	100
Colombia	1969	0.0	0.0	14.2	0.0	0.0	25.3	0.0	60.5	100
Colombia	2015.5	43.4	4.5	5.8	6.7	17.6	8.7	7.2	6.2	100
Costa Rica	1976	19.6	1.6	8.3	0.0	3.2	35.9	14.0	17.4	100
Costa Rica	2011	35.9	6.0	3.2	0.1	11.4	27.2	14.2	2.0	100
Cuba	1987	31.9	0.0	47.8	0.0	0.0	14.5	2.9	2.9	100
Cuba	2014	33.4	0.0	32.5	0.0	0.8	10.0	21.1	2.2	100
Dominican Rep.	1975	39.4	0.3	9.3	0.0	0.7	26.2	5.0	19.2	100
Dominican Rep.	2014	58.6	0.3	3.6	1.4	6.2	25.6	2.0	2.2	100
Ecuador	1979	24.3	0.6	15.0	0.0	2.8	29.6	3.1	24.6	100
Ecuador	2009.5	40.8	0.4	6.4	7.3	13.6	14.0	6.8	10.6	100
El Salvador	1985	67.5	1.5	7.0	0.0	1.5	14.0	2.5	5.9	100
El Salvador	2014	51.7	0.4	2.8	0.3	28.0	5.6	5.6	5.6	100
Guatemala	1977.5	33.3	2.3	7.3	0.0	6.2	30.5	4.0	16.4	100
Guatemala	2014.5	34.8	1.0	2.5	3.1	27.5	5.5	6.3	19.4	100
Haiti	1977	1.1	1.1	2.7	0.0	0.0	17.6	5.9	71.8	100
Haiti	2016.5	3.6	0.6	0.3	7.4	61.7	7.1	11.9	7.4	100
Honduras	1981	30.5	0.8	9.2	0.0	1.1	44.7	1.1	12.6	100
Honduras	2011.5	30.5	0.4	9.3	0.0	24.8	16.3	5.9	12.9	100
Jamaica	1975.5	22.0	0.0	5.4	0.0	16.8	32.2	17.9	5.7	100
Jamaica	2008.5	13.9	0.0	1.4	1.0	19.1	24.0	35.0	5.7	100
Mexico	1976.5	9.3	0.7	19.7	0.0	5.9	37.2	2.8	24.5	100
Mexico	2015	49.6	1.8	19.8	6.3	6.2	4.5	8.9	2.9	100
Nicaragua	1981	26.7	0.4	8.6	0.0	5.3	39.5	3.0	16.5	100
Nicaragua	2011.5	37.5	0.5	4.4	0.0	32.7	14.2	6.8	3.8	100
Panama	1975.5	40.7	0.8	7.1	0.0	1.2	32.6	2.3	15.4	100
Panama	2013	40.2	0.8	3.9	0.3	26.2	18.3	5.9	4.3	100
Paraguay	1977	11.6	0.0	14.4	0.0	3.2	42.6	9.4	18.8	100
Paraguay	2016	13.0	0.1	9.0	0.0	31.5	28.1	15.4	2.8	100
Peru	1969.5	8.0	0.0	4.0	0.0	0.0	12.0	12.0	64.0	100
Peru	2016	11.3	0.5	3.2	1.7	25.1	11.3	17.9	28.9	100
Puerto Rico	1968	57.0	2.3	2.7	0.0	0.0	18.9	3.5	15.6	100
Puerto Rico	1995.5	59.7	4.6	1.3	0.0	0.0	12.7	8.4	13.3	100
Trinidad and Tobago	1970.5	4.9	0.2	7.3	0.0	0.0	41.5	23.8	22.3	100
Trinidad and Tobago	2011	19.9	0.3	4.3	1.0	6.8	27.7	33.2	6.8	100
Uruguay	1986	0.0	0.0	13.0	0.0	0.0	57.2	15.4	14.3	100
Uruguay	2015	11.5	0.8	14.1	1.5	0.9	39.1	30.5	1.6	100
Venezuela*	1977	15.9	0.0	17.9	0.0	0.0	31.9	10.1	24.2	100
**NORTH AFRICA & WEST ASIA**										
Algeria	1986.5	0.0	0.0	6.2	0.0	0.0	78.9	1.5	13.4	100
Algeria	2012.5	0.9	0.0	4.0	0.4	0.2	77.5	3.4	13.7	100
Armenia	1991	0.0	0.0	13.9	0.0	0.0	3.0	28.0	55.0	100
Armenia	2015.5	1.2	0.0	15.8	0.0	0.4	4.6	26.1	51.9	100
Azerbaijan	2000	0.9	0.2	17.2	0.0	0.7	6.1	3.9	71.0	100
Azerbaijan	2006	0.8	0.0	18.5	0.0	0.0	2.2	4.4	74.0	100
Bahrain	1989	13.4	0.0	3.0	0.0	0.0	24.4	15.3	43.8	100
Bahrain	1995	10.2	0.0	4.8	0.0	0.0	17.9	15.8	51.3	100
Egypt	1974.5	0.0	0.0	10.1	0.0	0.0	80.2	0.0	9.7	100
Egypt	2014	2.1	0.0	51.5	0.9	14.6	27.4	0.9	2.7	100
Georgia	1999.5	4.1	0.0	24.7	0.0	0.0	2.5	16.0	52.7	100
Georgia	2010	5.6	0.0	24.2	0.0	0.0	7.9	26.4	35.9	100
Iran	1976.5	0.0	0.0	4.3	0.0	0.0	52.7	12.2	30.8	100
Iran	2010.5	18.0	3.5	10.2	0.0	4.4	19.0	17.4	27.4	100
Iraq	1974	4.4	0.0	4.4	0.0	4.4	64.0	10.3	12.5	100
Iraq	2011	6.2	0.0	18.9	0.2	6.2	31.7	3.6	33.1	100
Jordan	1972	0.0	0.0	4.0	0.0	0.0	57.1	4.5	34.4	100

Jordan	2012	3.7	0.0	35.7	0.5	1.5	13.6	13.3	31.7	100
Kuwait	1984.5	4.1	0.0	12.6	0.0	0.0	79.0	0.0	4.4	100
Kuwait	1999	7.9	0.0	16.9	0.0	0.0	44.9	5.6	24.8	100
Lebanon	1971	1.9	0.0	1.9	0.0	0.0	26.4	13.2	56.6	100
Lebanon	2009	1.3	0.0	33.3	0.0	0.0	42.4	8.2	14.7	100
Libya	1995	0.0	0.0	27.7	0.0	0.0	23.8	0.0	48.5	100
Libya	2007	3.6	0.0	16.3	0.0	0.7	22.3	5.5	51.6	100
Morocco	1979.5	4.1	0.0	8.2	0.0	0.0	70.6	1.5	15.5	100
Morocco	2010.5	3.7	0.0	6.5	0.0	1.5	74.7	0.0	13.6	100
Oman	1988.5	25.6	0.0	17.4	0.0	3.5	27.9	12.8	12.8	100
Oman	2014	11.8	0.7	9.4	0.3	13.6	19.2	7.0	38.0	100
Papua New Guinea	1996.5	29.3	0.8	0.4	0.0	26.3	17.0	1.9	24.3	100
Papua New Guinea	2006.5	26.6	1.5	0.0	0.0	28.2	14.2	4.3	25.1	100
Saudi Arabia	1996.5	3.2	0.0	20.9	0.0	0.6	62.0	2.8	10.4	100
Saudi Arabia	2016	1.6	0.0	17.6	0.0	0.8	62.0	2.9	15.1	100
Syria	1978	1.8	0.4	3.3	0.0	1.8	63.6	3.6	25.5	100
Syria	2009	4.8	0.0	42.5	0.0	1.7	16.6	3.9	30.5	100
Tunisia	1978	24.4	0.0	28.3	0.0	0.3	21.2	3.9	21.8	100
Tunisia	2011.5	5.0	0.0	41.1	0.7	1.6	30.9	1.8	18.9	100
Turkey	1963	0.0	0.0	0.0	0.0	0.0	3.8	16.3	79.8	100
Turkey	2013	12.8	0.0	23.0	0.0	0.8	6.3	21.6	35.5	100
United Arab Emirates*	1995	15.3	0.4	13.5	0.0	5.5	43.6	7.3	14.5	100
Yemen	1979	11.1	11.1	5.6	0.0	5.6	55.6	5.6	5.6	100
Yemen	2013	7.8	0.3	20.0	2.0	14.2	39.3	1.7	14.6	100
**SUB-SARAHAN AFRICA**										
Angola	1996	0.0	0.0	8.6	0.0	14.8	23.5	3.7	49.4	100
Angola	2015.5	0.7	0.0	1.5	5.2	35.1	26.1	23.1	8.2	100
Benin	1981.5	0.0	1.5	1.5	0.0	0.0	4.5	3.0	89.4	100
Benin	2014	1.2	0.0	6.2	16.8	22.4	16.1	7.5	29.8	100
Botswana	1984	5.4	0.0	17.3	0.0	3.6	36.1	4.3	33.2	100
Botswana	2007.5	3.5	0.2	1.3	0.0	11.3	10.1	69.3	4.3	100
Burkina Faso	1992.5	0.8	0.0	2.8	0.0	0.4	8.5	3.3	84.1	100
Burkina Faso	2016.5	0.0	0.0	3.5	46.5	32.3	11.0	3.1	3.5	100
Burundi	1987	1.1	0.0	3.4	0.0	5.7	2.3	1.1	86.2	100
Burundi	2016.5	1.8	0.4	3.3	21.7	42.0	6.2	4.3	20.3	100
Cameroon	1978	0.0	0.0	8.3	0.0	0.0	8.3	8.3	75.0	100
Cameroon	2014	0.7	0.0	1.0	4.5	15.4	7.9	25.0	45.5	100
Central African Rep.	1994.5	2.7	0.0	0.7	0.0	4.1	7.5	6.8	78.2	100
Central African Rep.	2010.5	1.6	0.0	0.0	1.6	4.1	48.4	18.9	25.4	100
Chad	1996.5	4.8	0.0	0.0	0.0	4.8	14.3	4.8	71.4	100
Chad	2014.5	4.3	0.0	0.0	21.7	45.7	8.7	6.5	13.0	100
Congo	2005	0.5	0.0	0.2	0.0	2.3	5.3	20.6	71.0	100
Congo	2014.5	0.7	0.0	0.0	1.4	10.1	16.4	31.0	40.4	100
Congo, Democatic Rep.	1983	4.7	0.0	2.7	0.0	3.1	5.4	1.2	82.9	100
Congo, Democatic Rep.	2013.5	3.5	0.5	1.0	3.5	6.0	3.5	17.1	64.8	100
Côte d'Ivoire	1980.5	0.0	0.0	3.4	0.0	0.0	13.8	0.0	82.8	100
Côte d'Ivoire	2016	1.4	0.7	0.7	7.7	36.6	44.4	0.0	8.5	100
Equatorial Guinea	2000	5.4	1.1	7.6	1.1	25.0	16.3	4.3	39.1	100
Equatorial Guinea	2011	8.9	0.0	4.5	0.0	18.8	20.5	19.6	27.7	100
Eritrea	1995.5	3.8	0.0	7.5	0.0	10.0	25.0	3.8	50.0	100
Eritrea	2010	3.0	0.0	4.5	0.0	26.9	34.3	11.9	19.4	100
Ethiopia	1990	6.3	0.0	6.3	0.0	0.0	45.8	2.1	39.6	100
Ethiopia	2017	0.6	0.0	3.3	22.9	64.4	5.0	0.3	3.6	100
Gabon	2000	3.2	0.0	0.0	0.0	1.6	15.6	16.6	63.0	100
Gabon	2012	2.0	0.0	0.0	0.0	1.3	18.8	39.5	38.5	100
Gambia	1990	3.4	0.0	7.6	0.0	14.4	28.0	3.4	43.2	100
Gambia	2013	6.7	0.0	3.3	6.7	43.3	23.3	6.7	10.0	100
Ghana	1979.5	6.3	0.0	3.8	0.0	1.3	30.4	7.6	50.6	100
Ghana	2017	4.9	0.0	2.0	27.6	25.7	15.8	5.6	18.4	100
Guinea	1992	0.0	0.0	0.0	0.0	0.0	38.5	7.7	53.8	100
Guinea	2016	0.0	0.0	0.0	9.6	42.3	32.7	0.0	15.4	100
Guinea-Bissau	2000	4.5	0.0	34.3	0.0	7.5	4.5	1.5	47.8	100
Guinea-Bissau	2014	1.5	0.0	26.9	25.4	10.8	11.5	11.5	12.3	100

Kenya	1977.5	14.5	0.0	10.1	0.0	8.7	29.0	1.4	36.2	100
Kenya	2016	4.9	0.0	5.8	29.8	45.5	8.4	3.0	2.6	100
Lesotho	1977	15.3	0.0	2.8	0.0	4.2	22.2	2.8	52.8	100
Lesotho	2014	2.8	0.2	2.2	2.3	40.0	23.7	28.2	0.7	100
Liberia	1986	17.7	0.0	9.7	0.0	4.8	53.2	0.0	14.5	100
Liberia	2016	3.0	0.0	1.0	11.6	62.8	16.3	4.3	1.0	100
Madagascar	1992	5.5	0.0	3.0	0.0	9.7	8.5	3.0	70.3	100
Madagascar	2012.5	3.6	0.0	1.8	6.7	51.6	17.1	2.6	16.6	100
Malawi	1984	0.0	0.0	6.3	0.0	2.1	14.6	0.0	77.1	100
Malawi	2015.5	18.5	0.2	1.9	19.5	50.8	4.1	3.2	1.9	100
Mali	1987	2.2	0.0	2.2	0.0	2.2	19.6	0.0	73.9	100
Mali	2015	1.9	0.0	1.3	28.6	41.6	24.0	0.0	2.6	100
Mauritania	1981	28.6	0.0	0.0	0.0	0.0	0.0	0.0	71.4	100
Mauritania	2015	0.6	0.0	1.7	4.5	20.1	59.8	1.1	12.3	100
Mauritius	1985	6.2	0.0	3.1	0.0	8.6	28.3	12.5	41.2	100
Mauritius	2014	11.6	0.0	2.6	1.4	2.6	14.2	16.9	50.7	100
Mozambique	1997	12.7	0.0	5.5	0.0	41.8	25.5	5.5	9.1	100
Mozambique	2015	0.8	0.0	3.1	6.6	51.9	24.8	5.8	7.0	100
Namibia	1989	15.6	0.2	3.4	0.0	39.1	20.0	0.0	21.5	100
Namibia	2013	11.7	0.5	2.2	0.4	49.0	12.8	21.9	1.5	100
Niger	1992	2.2	0.0	4.4	0.0	11.1	33.3	0.0	48.9	100
Niger	2017	0.5	0.0	1.1	16.5	38.8	38.8	0.0	4.3	100
Nigeria	1981.5	0.0	0.0	1.5	0.0	3.0	7.5	0.0	88.1	100
Nigeria	2016.5	1.6	0.0	5.6	11.1	34.1	18.3	8.7	20.6	100
Qatar	1987	14.2	0.0	27.0	0.0	0.0	41.2	6.9	10.7	100
Qatar	2012	2.2	3.0	29.2	0.5	11.7	36.2	8.7	8.4	100
Rwanda	1983	0.0	0.0	2.7	0.0	3.6	1.8	0.0	91.8	100
Rwanda	2014.5	2.3	0.4	2.1	14.8	46.0	16.1	7.3	11.1	100
Senegal	1978	0.0	0.0	5.1	0.0	0.0	7.7	2.6	84.6	100
Senegal	2016	2.8	0.0	6.4	28.5	32.9	18.5	2.4	8.4	100
Sierra Leone	1992	0.0	0.0	16.0	0.0	8.0	68.0	4.0	4.0	100
Sierra Leone	2013	3.2	0.0	0.6	15.4	48.1	25.0	1.3	6.4	100
Somalia	1999	0.0	0.0	0.0	0.0	0.0	50.0	0.0	50.0	100
Somalia	2006	0.0	0.0	5.9	0.0	11.8	47.1	0.0	35.3	100
South Africa	1988	16.2	2.8	10.7	0.0	39.7	26.7	1.4	2.4	100
South Africa	2016	14.1	1.1	2.2	6.1	43.9	15.4	16.1	1.1	100
South Sudan	2006	0.0	0.0	0.0	0.0	0.0	16.7	66.7	16.7	100
South Sudan	2010	2.9	0.0	0.0	0.0	11.4	8.6	11.4	65.7	100
Sudan	1978.5	6.7	0.0	2.2	0.0	4.4	68.9	2.2	15.6	100
Sudan	2014	0.0	0.0	3.4	2.6	12.1	77.6	0.0	4.3	100
Swaziland	1988.5	16.2	1.0	9.1	0.0	27.9	27.9	3.6	14.2	100
Swaziland	2014	5.7	0.0	1.1	5.8	33.6	16.1	36.7	0.9	100
Tanzania	1991.5	15.4	0.0	3.8	0.0	3.8	32.7	6.7	37.5	100
Tanzania	2015.5	8.9	0.3	2.4	17.6	33.2	14.5	6.3	16.8	100
Togo	1988	1.8	0.0	2.4	0.0	0.6	1.2	1.2	92.8	100
Togo	2013.5	1.5	0.0	4.0	23.7	35.9	11.1	10.6	13.1	100
Uganda	1988.5	16.3	0.0	4.1	0.0	8.2	22.4	0.0	49.0	100
Uganda	2017	6.9	0.0	2.4	18.8	46.7	7.2	6.1	11.9	100
Zambia	1992	13.9	0.0	3.3	0.0	0.7	28.5	11.9	41.7	100
Zambia	2013.5	4.0	0.0	2.5	11.4	40.1	24.5	8.3	9.1	100
Zimbabwe	1984	4.2	0.3	1.8	0.0	2.1	59.0	1.8	30.8	100
Zimbabwe	2015	1.2	0.0	0.9	14.5	14.5	61.7	5.7	1.5	100
